# Supplemental Breast Ultrasound in Mammography Screening for Women with Critically Dense Breasts

**DOI:** 10.3390/cancers18101631

**Published:** 2026-05-19

**Authors:** Sylvia H. Heywang-Köbrunner, Susanne A. Elsner, Eva Haußmann, Astrid Hacker, Paula Grieger, Moritz Hadwiger, Michael Hertlein, Alexander Katalinic

**Affiliations:** 1 Referenzzentrum Mammographie München, Sonnenstraße 29, 80331 Munich, Germany; 2 FFB gGmbH, Sonnenstraße 29, 80331 Munich, Germany; 3 Institut für Sozialmedizin und Epidemiologie, Universität zu Lübeck, Ratzeburger Allee 160, 23562 Lübeck, Germany (M.H.); alexander.katalinic@uksh.de (A.K.)

**Keywords:** mammography, ultrasonography, mammary, early detection of cancer, risk assessment, program evaluation, breast density, breast neoplasms

## Abstract

**Background:** The sensitivity of mammography screening is compromised in women with dense breast tissue. Supplemental ultrasound (S-US) can enhance cancer detection, but evidence regarding its feasibility, harms, and benefits within Western population-based screening programs remains limited. **Methods:** This pragmatic, prospective controlled trial was integrated into the German national mammography screening program across 16 sites. Breast density was assessed using automated AI software. Women with “critically dense” breasts (top 15–20%) were offered handheld supplemental S-US in addition to mammography (MXUS). The control group (MX-only) comprised women with comparable densities who did not receive S-US. Primary outcomes included cancer detection rate (CDR), recall rate, biopsy rate, and short-term follow-up recommendations. **Results:** From May 2020 to March 2024, 25,341 women underwent MXUS, while 38,529 received MX-only. The CDR was significantly higher in the MXUS group, at 10.7 per 1000 (95% CI: 9.4–12.0) compared to 7.2 per 1000 (95% CI: 6.4–8.1) in the MX-only group, yielding an incremental CDR of 3.5 per 1000 (95% CI: 1.9–5.0). However, MXUS led to higher rates of recall (6.6% vs. 5.4%), biopsies (3.2% vs. 1.5%), and short-term follow-up recommendations (0.9% vs. 0.5%). **Conclusions:** Implementing quality-assured S-US for women with critically dense breasts substantially increases the detection of invasive cancers, but also raises false positives. While these results support density-adapted screening, the high resource intensity suggests that future strategies should optimize risk stratification to target S-US more selectively. Long-term data are required to confirm clinical benefits.

## Simple Summary

International recommendations for or against supplementary breast cancer screening for women at average risk with mammographically dense tissue diverge. Decisions for or against supplemental screening or selection of appropriate methods require evidence on their potential advantages, harms, and feasibility. A pragmatic controlled trial was performed prospectively in 63,870 participants of the German population-based screening program, comparing mammography plus ultrasound versus mammography alone. Important screening quality parameters were systematically recorded for the independent intervention and control groups. Both groups were selected from the subgroup of women with the highest 15–20% of AI-derived breast density values (i.e., critically dense tissue). In the intervention group, the cancer detection rate increased substantially (+3.5/1000). Recall, biopsy, and short-term follow-up rates increased less than expected. Feasibility was challenging. Biological significance of the detected malignancies can be assessed after an adequate follow-up period.

## 1. Introduction

Women with dense breast tissue benefit less from population-based mammography screening (MSP) than women with non-dense breasts. High breast density reduces the sensitivity of mammography by obscuring tumors and is also an independent risk factor for breast cancer [1,2]. Within European screening programs, approximately 5–10% of women have mammographic breast tissue density classified as extremely dense (ACR category D) and another 30–40% as heterogeneously dense (ACR category C). With increasing breast density, program sensitivity steadily decreases, from 75–86% with ACR A/B to 51–71% with ACR D, while interval cancer rates increase [3,4,5].

To address the lower sensitivity of mammography in dense breast tissue, supplemental imaging has been proposed and is widely used in opportunistic screening worldwide. Ultrasound (US) is the most commonly used supplementary imaging modality, and numerous studies have shown that its addition increases cancer detection compared with mammography alone [6,7,8,9,10,11]. Unfortunately, side effects of supplementary US such as additional recall, biopsies, or short-term follow-up recommendations have been reported inconsistently, and wide variations exist between different studies. Overall, the additional detection of supplementary US comes at the cost of significant increases in false-positive recalls and biopsies [6,11,12,13,14], and the overall balance between benefit and harm remains controversial [13]. Some studies and reviews have even suggested that the harms of supplementary imaging may outweigh the benefits [14].

Accordingly, international recommendations vary considerably. A recent systematic review of guidelines highlighted substantial heterogeneity, ranging from no recommendation for supplemental screening to endorsement of US, MRI, or tomosynthesis [15]. Guidelines discouraging supplemental screening cite increased false positives, potential overdiagnosis, and insufficient evidence of mortality benefit. A retrospective study from the US reported that supplemental screening is inconsistently applied and often depends on provider preference or state-level density notification laws [16].

The only randomized controlled study, the Japanese J-START trial [7,10], showed that S-US detected 2–3 additional cancers per 1000 women and reduced interval cancers by 50%, but specificity decreased substantially. Generalizability to European programs remains uncertain, given the younger age range (40–49 years) and differences in breast size and tissue composition between Asian and European women. Systematic reviews emphasized that most observational studies outside Asia are small, heterogeneous, and lack embedding in population-based programs [6]. Large-scale, real-world data in unselected screening populations reflecting the demographics and operational realities of Western screening strategies are therefore urgently needed.

Against this background, we investigated the feasibility and diagnostic outcomes of supplemental ultrasound within the German MSP, Europe’s largest invitation-based, quality-assured screening program. We focused on the 15–20% of women with the highest breast densities, comprising all ACR D and the densest subset of ACR C. This range was defined as “critically dense” breast tissue. It was chosen because offering S-US to all women with ACR C or D breasts (accounting for 40–50% of the population) was not considered operationally feasible, whereas limiting S-US to critically dense tissue represents a more practical and targeted approach. We hypothesized that the addition of handheld ultrasound (HH-US) in this subgroup would result in the detection of at least two additional cancers per 1000 screened women. Our study aimed to systematically compare cancer detection, recall, short-term follow-up, and biopsy rates between women with critically dense breast tissue undergoing mammography plus ultrasound (MXUS) and those receiving mammography alone (MX-only).

## 2. Materials and Methods

### 2.1. Study Design and Participants

The study was conducted within the framework of the German national MSP, an organized, population-based, certified program that follows European guidelines for mammography screening [17]. The German program targets women at average risk of breast cancer, while women at higher risk, such as those with a genetic predisposition, receive tailored screening recommendations. All eligible women are invited biennially by mail. Invitations are sent through a central invitation office, which obtains address data from the state residents’ registration offices in accordance with data protection regulations. During the study period, women aged 50 to 69 years were eligible for invitation to the MSP. The MSP includes double reading, consensus conferences, and further assessment in the case of positive findings. Screening participants are typically not informed about their breast density.

DIMASOS2 is a pragmatic, non-randomized controlled trial. Between May 2020 and March 2024, 16 screening sites across Germany identified potential participants. Eligibility required mammographic density within the top 15% to 20% of the distribution. This density range of 15–20% was a compromise between feasibility and group size. Exclusion criteria included breast implants, participation in other diagnostic breast imaging trials, language barriers, or lack of written informed consent. Participating screening sites prospectively allocated fixed timeslots for recruitment and non-recruitment. These timeslots were not communicated to patients or the invitation office. Density was measured continuously in all mammograms to identify women with critically dense breasts. During recruitment timeslots, eligible women were informed about the study and invited for supplemental HH-US. Those who underwent S-US formed the intervention group (MXUS group).

Randomized allocation of study participants to mammography plus S-US or mammography alone was deliberately avoided because of the high risk of contamination. Women assigned to the control group would likely have sought additional US after receiving information on their breast tissue density. To minimize this bias, women undergoing mammography during non-recruitment timeslots were neither informed of the density measurement nor asked to provide written consent. These women comprised the control group (MX-only group), and their routinely documented screening and outcome data were anonymized for analysis.

The sample size was calculated to detect 2 additional tumors per 1000 participants in the MXUS group, with a precision of 0.55 per 1000, resulting in 25,000 screened participants. The MX group was not size-limited, in order to achieve the highest possible statistical power.

### 2.2. Procedures

Automated breast density measurement was prospectively integrated into routine screening using the CE-certified AI-based software Densitas Density Software 2.5.0™ (Densitas, Halifax, NS, Canada). The software provides density measurements on a continuous scale from 1 to 100. Density thresholds identifying the top 15–20% were defined for each mammography unit and vendor, based on 17,628 previously analyzed screening mammograms from participating sites. Participants with scores above the threshold were classified as having critically dense breasts and were flagged by the study software.

Eligibility for study participation was determined immediately after the mammographic examination, and radiographers were notified by an automated pop-up message. Eligible women were required to sign informed consent before they were offered an appointment for supplemental HH-US (MXUS group) within one week of the initial mammography. During non-recruitment slots, no pop-up appeared. Thus, potentially eligible women examined during these time slots were not informed and comprised the MX-only group.

Since these women underwent usual care, while their data was only available anonymized, no informed consent was legally required. Both the MXUS and MX-only groups underwent mammography according to German MSP regulations, with craniocaudal and mediolateral oblique mammographic views. In line with the mandatory national screening guideline, all mammograms were independently and blindly evaluated by two certified, experienced screen readers.

Participating screen readers of DIMASOS2 were required to demonstrate proficiency in HH-US, including former training and >1000 US examinations during the last 3 years. They also completed a mandatory training workshop before study initiation. Only upper-middle and high-end US devices that passed stringent entrance testing criteria were authorized for DIMASOS2. S-US was performed by physicians, and the same screen readers interpreted MX and S-US jointly.

The second reader evaluated the mammogram and the recorded US images, blinded to the first reader’s findings. If either reader identified positive findings, the case was reviewed in a consensus conference (head of screening unit and readers). Women with positive consensus findings were recalled for further assessment, which could include additional mammographic views, tomosynthesis, bilateral breast US with or without Doppler or elastography, and rarely contrast-enhanced mammography or MRI. Women with probably benign findings (BI-RADS category 3) after imaging assessment were invited for short-term follow-up, typically at six months. For positive findings (BI-RADS 4 or 5), histopathological assessment, usually percutaneous biopsy, was performed, and results were reviewed in multidisciplinary conferences. Postoperative data were collected for all histopathologically confirmed malignancies. All data were prospectively documented in the official screening database. These data, along with breast density measurements, were automatically transferred to the study database. Data from the MX-only group were available only as individual anonymized files, whereas for the MXUS group, US results were entered directly into the study database and outcomes were transmitted to the screening software.

### 2.3. Outcomes

The primary outcome was the breast cancer detection rate. Breast cancer was defined as histopathologically confirmed invasive or non-invasive cancer following a screening-initiated biopsy. Secondary outcomes included recall, short-term follow-up, and biopsy rates. Additionally, positive predictive values (PPV) were evaluated. PPV1 is defined as the proportion of detected cancers among participants with a positive consensus finding, and PPV2 as the proportion of detected cancers among women undergoing biopsy. The histopathological characteristics of the detected cancers included tumor grade, size (mm), and stage, classified according to UICC/TNM classification, 8th edition.

### 2.4. Statistical Analysis

Descriptive statistics were used to summarize baseline characteristics, with continuous variables reported as medians (interquartile range, IQR) and categorical variables as frequencies (%). Density values from different devices were rescaled to a uniform scale (50–100). Due to the observational nature of the study and the observed differences in baseline characteristics between the study groups, stabilized inverse-propensity treatment weighting (sIPTW) was applied. Propensity scores were estimated via logistic regression, including possible confounders like age, breast density score, region, and screening round. Weights were derived to generate a sample with balanced covariates. The balance of the covariates was assessed before and after weighting using standardized mean differences and propensity score distributions (Supplement Figure S1). sIPTW-adjusted outcome rates were expressed per 1000 women or as percentages, with 95% confidence intervals (CIs). Absolute risk differences and risk ratios (RRs) were calculated to quantify differences between the groups. Cancer characteristics were compared using *t*-tests or Wilcoxon rank-sum tests for continuous data and chi-squared or Fisher’s exact tests for categorical data. Two-sided *p* values < 0.05 were considered statistically significant. All analyses were conducted using R (version 4.1.3), with sIPTW performed via the WeightIt package [18]. The R code underwent internal peer review.

## 3. Results

**Figure 1:**
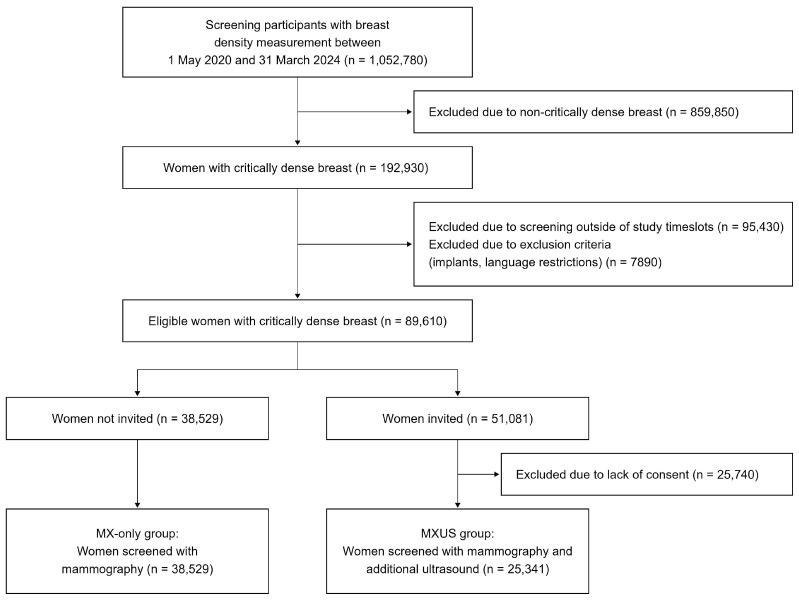
Flow diagram of screening participants, exclusions, and allocation to the MX-only and MXUS groups.

In total, 1,052,780 participants of the MSP, aged 50–69 years, underwent initial breast density measurement (Figure 1). Of these, 192,930 (18.3%) were flagged as having critically dense breast tissue. After excluding those outside recruitment time slots, with implants, language restrictions, or without consent, the study population comprised 63,870 women. Of these, 25,341 underwent S-US (MXUS group) following screening mammography, and 38,529 received screening mammography only (MX-only group).

The baseline characteristics are described in Table 1. Women in the MXUS group were younger (median age in years: 55 vs. 56), had higher breast density scores (59 vs. 57), and the proportion of first-round screened women was higher (31% vs. 27%), reflecting the higher density range in younger women. The proportion of women with a previous history of breast cancer was 0.4% in both groups.

Overall, 549 breast cancer cases were detected in the study population, of which 26.6% were diagnosed as DCIS. Crude observed cancer detection rate, recall, short-term follow-up, and biopsy rates are presented in Supplement Table S1. The model-based cancer detection rate was 10.70 per 1000 women (95% CI 9.43–11.97) in the MXUS group and 7.24 (95% CI 6.39–8.09) in the MX-only group (Table 2). This corresponds to 3.46 (95% CI 1.93–4.98) additional detected breast cancer per 1000 women in the MXUS group, representing a 48% increase in breast cancer detection (RR 1.48 (95% CI 1.25–1.74)). The cancer detection rate for DCIS was comparable in both groups; however, the cancer detection rate of invasive cancers in the MXUS group was 67% (RR 1.67 (95% CI 1.37–2.03)) higher compared to the MX-only group (8.29 vs. 4.97 per 1000).

Recall occurred in 6.59% of the participants in the MXUS group, compared to 5.36% in the MX-only group (Table 2), representing a relative increase of 23% (RR 1.23 (95% CI 1.16–1.31)). Comparing the MXUS and MX-only group, short-term follow-up almost doubled (0.93% vs. 0.51%; RR 1.82 (95% CI 1.50–2.20)) and the biopsy rate in the MXUS group was more than twice as high (3.16% vs. 1.47%; RR 2.16 (95% CI 1.94–2.40)).

PPV1 increased by 2.72% (16.2% vs. 13.5%) for the MXUS group compared to MX-only, while PPV2, the rate of malignancy among biopsies, was nearly one-third lower for MXUS (33.9% vs. 49.4%) (Table 2).

Women with detected breast cancer in the MXUS group were approximately 3 years older than those in the MX-only group (Table 3, Supplement Table S2). Despite the higher cancer detection rate in the MXUS group, no statistically significant differences were found in tumor-related parameters between the groups (Table 3).

**Figure 2:**
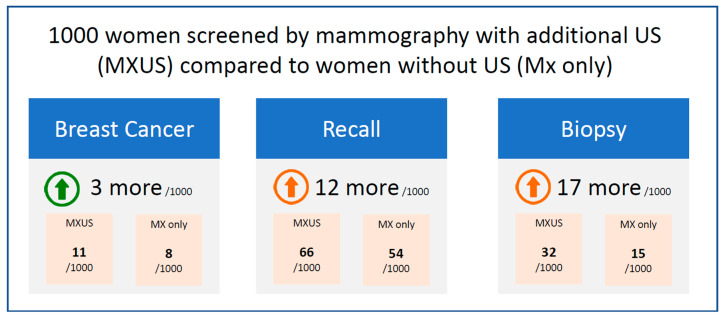
Summary of findings, numbers have been rounded up to the next nearest whole number. green arrow: desirable effect; red arrow: undesirable side-effect.

Benefits and harms are compared in Figure 2. To detect one additional breast cancer by MXUS, almost 300 women must undergo S-US; one additional biopsy occurs for every 60 screened women, and approximately five additional biopsies are needed to detect one additional cancer.

## 4. Discussion

To our knowledge, this is the largest multicenter cohort study on supplemental ultrasound (S-US) in women with dense breast tissue conducted within a national, population-based screening program. We observed a 48% increase in overall breast cancer detection rate with mammography combined with ultrasound (MXUS) compared with mammography alone (MX-only), corresponding to an additional 3.5 cancers per 1000 women screened. This increase was almost entirely attributable to invasive cancers (+67%), while DCIS detection was similar in both groups.

Further, although the stage distribution of invasive tumors is largely similar between the two groups, the absolute rate of prognostically favorable tumors (T1) increased by more than 60% (from 3.89 per 1000 to 6.33 per 1000). These additionally detected small tumors can generally be managed with less invasive, more conservative treatment. Without this detection, they would likely have been diagnosed later as interval cancers—presumably at a less favorable stage and requiring more intensive therapy.

Alongside the higher cancer detection rate, recall, short-term follow-up, and biopsy rates increased, PPV1 improved modestly, while PPV2 declined.

### 4.1. Comparison with Previous Evidence

Our results align with findings from the Japanese J-START trial, the only randomized controlled trial (RCT) of S-US, which included 72,717 women aged 40–49 years irrespective of breast density. J-START demonstrated increased detection of invasive cancers and a stage shift towards earlier-stage disease [10]. For a subgroup of women with dense tissue within the J-START trial (11,390 with ACR C or D densities among 19,213 women), Harada-Shoji et al. reported 1–3 additional cancers per 1000 women, also consistent with our findings [7]. Transferability of these results to European screening populations has been questioned due to differences in breast size, density distribution, pattern, and age. Nevertheless, the effect size in our study is comparable.

In women with average to intermediate risk, observational studies in Europe and North America have shown comparable additional cancer detection rates, ranging from 2 to 4.4 cancers per 1000 women [6,8,9,11,13], and Rebolj et al. estimated that up to 40% of screen-detected cancers in dense tissue could be attributed to ultrasound alone. Systematic reviews [6,9,11,13] confirmed that ultrasound improves cancer detection but leads to significantly higher recall and biopsy rates. All reviews, however, emphasized the lack of evidence on subsequent outcomes, such as interval cancer rates or mortality reduction.

Notably, the overall cancer detection rate in our study was higher than that reported in the most recent meta-analysis [6]. This higher detection rate may reflect differences in population characteristics, screening protocols, and quality assurance of MXUS.

### 4.2. Interpretation of Benefits and Harms

The balance of benefits and harms is central to any screening intervention. In our study, detecting one additional cancer required ultrasound examinations in approximately 300 women, resulting in one additional biopsy for every 60 women screened, and approximately five additional biopsies to detect one additional cancer. These numbers highlight the substantial burden imposed on healthy women, who represent >99% of the screened population. Although PPV1 improved modestly, the significant reduction in PPV2 underscores the increased number of false-positive biopsies.

Importantly, increased cancer detection is a prerequisite for achieving better outcomes. However, improved detection alone does not guarantee improved clinical outcomes. Overdiagnosis remains a concern, particularly if supplemental imaging leads to very high cancer detection rates. Nevertheless, the similar distribution of tumor size, stage, and grade across groups suggests that MXUS predominantly identified clinically important invasive cancers that would otherwise have been missed. The increased detection of cancers at favorable stages, sizes, and grades offers promising prognostic impact and supports the evaluation of follow-up data. However, definitive conclusions require follow-up analyses of interval cancers and long-term outcomes. The design of this study, embedded in the German MSP with planned cancer registry linkage, provides an opportunity to evaluate the prognostic significance of the additional cancers detected.

### 4.3. Feasibility and Resource Implications

Implementation of S-US proved logistically demanding [19]. Examination times of 15–25 min per woman (compared to 1 min for MX-only) imposed substantial time constraints, and most study sites reported organizational difficulties. We estimate that the time invested by highly trained physicians per additional cancer detection was approximately 20 times higher for MXUS compared to standard mammography screening. These findings raise serious concerns regarding the feasibility of large-scale implementation of MXUS in population-based settings.

The resulting additional biopsies naturally led to an increased workload. For every 1000 screened women, we detected 3 more carcinomas, requiring 12 additional recalls and 17 more biopsies (see Figure 2).

Alternative approaches to improve feasibility include automated breast ultrasound (ABUS), which could reduce dependence on physician-performed examinations. Current evidence suggests that diagnostic performance of ABUS and handheld ultrasound (HH-US) is largely equivalent [20,21,22]. However, ABUS requires costly equipment and trained technologists, who are in short supply. Comparative trials on women at intermediate-to-high risk favored contrast-enhanced imaging over HH-US or ABUS [23,24] but did not include normal risk screening populations. In normal-risk populations, risk-stratified strategies that limit S-US to women with the densest breast categories and additional risk factors may improve feasibility. Emerging artificial intelligence (AI) approaches for density assessment and risk prediction based on mammographic, clinical and genetic features show promise for more targeted deployment of supplemental imaging [25,26,27,28].

### 4.4. Generalizability

Our results are directly applicable to screening programs that rely on full-field digital mammography (FFDM), the current standard in Germany and most European countries. Extrapolation to settings using digital breast tomosynthesis (DBT) is uncertain. DBT has demonstrated superior sensitivity compared to FFDM [29], and whether S-US provides additional benefit in DBT-based screening of women at normal risk requires further evaluation. Detection of DCIS was unaffected, as expected, due to mammography’s established strengths for detecting this entity.

### 4.5. Strengths and Limitations

This study is observational and, therefore, subject to bias and confounding. Contamination was minimized by blinding the control group to breast density, but self-selection bias in the intervention group cannot be excluded. The exclusive use of HH-US and the absence of DBT in the screening program limit generalizability.

Key strengths include the study’s embedding in a highly standardized national program with strict quality assurance, near-complete documentation, and a very large sample size. The combined MX + US reading was implemented in a controlled, quality-assured manner, reflecting real-world practice in a population-based program. In this study, an attempt was made to reduce observer dependence through specialized training.

## 5. Conclusions

This large real-world study demonstrates that supplemental ultrasound in women with dense breasts significantly increases invasive cancer detection but also raises recall and biopsy rates. Implementation was logistically demanding and resource-intensive, with examination times of 15–25 min and disproportionately high physician time per additional cancer detected. Our findings support the potential role of MXUS in density-adapted screening strategies but highlight the importance of feasibility and resource allocation. Long-term follow-up with cancer registry linkage will be essential to assess effects on interval cancers, stage distribution, and ultimately, breast cancer mortality. Further research on optimized stratification for supplementary imaging is crucial, even among women with dense breast tissue, before considering widespread implementation in population-based screening.

## Figures and Tables

**Table 1:** Characteristics of the study population by study group and overall.

Characteristics	MXUS Group (n = 25,341)	MX-Only Group (n = 38,529)	Overall (n = 63,870)
**Age, years**			
Median [IQR] ^1^	55 (52–60)	56 (52–61)	56 (52–61)
**Age groups, n (%)**			
50–54	12,224 (48.2)	16,495 (42.8)	28,719 (45.0)
55–59	6074 (24.0)	9553 (24.8)	15,627 (24.5)
60–64	4147 (16.4)	7133 (18.5)	11,280 (17.7)
65–69	2896 (11.4)	5348 (13.9)	8244 (12.9)
**Breast Density**			
Median [IQR]	59 (54–66)	57 (53–63)	58 (53–64)
**Screening round, n (%)**			
First	7963 (31.4)	10,370 (26.9)	18,333 (28.7)
Follow-up	17,378 (68.6)	28,159 (73.1)	45,537 (71.3)
**Region, n (%)**			
Urban	25,022 (98.7)	36,573 (94.9)	61,595 (96.4)
Rural	319 (1.3)	1956 (5.1)	2275 (3.6)
**History of breast cancer, n (%)**			
Yes	99 (0.4)	148 (0.4)	247 (0.4)
No	25,242 (99.6)	38,381 (99.6)	63,623 (99.6)

^1^ IQR: Interquartile Range.

**Table 2:** Adjusted primary and secondary outcome measures using stabilized inverse-propensity treatment weighting.

	MXUS Group (n = 25,341)	MX-Only Group (n = 38,529)	Absolute Risk Difference (ARD)	Relative Risk (RR) ^1^
**Cancer detection rate** per 1000 [95% CI]	10.70 [9.43; 11.97]	7.24 [6.39; 8.09]	3.46 [1.93; 4.98]	1.48 [1.25; 1.74]
**Ductal carcinoma in situ rate** per 1000 [95% CI]	2.40 [1.79; 3.00]	2.27 [1.79; 2.74]	0.13 [−0.64; 0.89]	1.06 [0.76; 1.46]
**Invasive cancer rate** per 1000 [95% CI]	8.30 [7.19; 9.42]	4.97 [4.27; 5.67]	3.33 [2.01; 4.65]	1.67 [1.37; 2.03]
**Recall rate** in % [95% CI]	6.59 [6.28; 6.89]	5.36 [5.13; 5.58]	1.23 [0.85; 1.61]	1.23 [1.16; 1.31]
**Short-term follow-up rate** in % [95% CI]	0.93 [0.81; 1.04]	0.51 [0.44; 0.58]	0.42 [0.28; 0.55]	1.82 [1.50; 2.20]
**Biopsy rate** in % [95% CI]	3.16 [2.94; 3.37]	1.47 [1.35; 1.59]	1.69 [1.45; 1.94]	2.16 [1.94; 2.40]
**PPV1 ^2^** in % [95% CI]	16.24 [14.27; 18.21]	13.52 [11.91; 15.12]	2.72 [0.18; 5.26]	1.20 [1.04; 1.42]
**PPV2 ^3^** in % [95% CI]	33.87 [29.76; 37.98]	49.43 [43.57; 55.29]	−15.55 [−22.71; −8.40]	0.69 [0.58; 0.81]

^1^ Relative Risk for MXUS with reference MX-only, ^2^ PPV1 = positive predictive value 1: proportion of detected cancers among participants with a positive finding following the consensus conference; ^3^ PPV2: proportion of detected cancers among those who underwent biopsy.

**Table 3:** Adjusted characteristics of the detected cancers using stabilized inverse-propensity treatment weighting, numbers have been rounded up to the next nearest whole number.

	MXUS Group	MX-Only	*p*-Value
**Age**			0.025
Median [IQR]	57 (52–62)	54 (51–60)	
**DCIS, %**	22.4	31.3	0.022
per 1000	2.40	2.27	
**Invasive breast cancers; %**	77.6	68.7	0.022
per 1000	8.30	4.97	
**T stage, % of invasive cancer**			0.203
T1 per 1000	76.2 6.33	78.3 3.89	
T2 per 1000	23.3 1.93	19.2 0.96	
T3/4 per 1000	0.5 0.04	2.5 0.12	
**UICC stage, % of invasive cancer**			0.373
Stage I per 1000	70.7 5.87	71.6 3.56	
Stage II per 1000	22.6 1.88	25.0 1.25	
Stage III–IV per 1000	4.5 0.37	2.9 0.15	
Stage missing per 1000	2.2 0.19	0.5 0.02	
**Histopathological grading, % of invasive cancer**			0.837
Grade 1	29.3	25.9	
Grade 2	57.5	59.8	
Grade 3	8.3	9.9	
Grade missing	5.0	4.5	
**Invasive cancer size, mm**			0.989
Median [IQR]	14 (9–20)	14 (9–18)	

## Data Availability

The study protocol is publicly available (https://t1p.de/0kx95; accessed on 1 April 2026). Anonymized participant data can be shared upon reasonable request to the corresponding author, subject to approval of a methodologically sound proposal and in line with data protection regulations. All statistical code used for the analyses is archived and freely accessible via OFS (https://t1p.de/532l7; accessed on 1 April 2026).

## Supplementary Materials

The following supporting information can be downloaded at: https://www.mdpi.com/article/10.3390/cancers18101631/s1, Figure S1: Covariate balance before and after sIPTW; A: Love plot of absolute mean differences for all covariates before and after weighting; B: Density plot of the propensity score for the MX-only group (Treatment = 0) and the MXUS group (Treatment = 1) before and after weighting., Table S1: Observed primary and secondary outcome measures, Table S2: Observed characteristics of the detected cancers.

## Abbreviations

The following abbreviations are used in this manuscript:

CI	Confidence interval
DBT	digital breast tomosynthesis
DCIS	ductal carcinoma in situ
FFDM	Full-field digital mammography
HH-US	handheld breast ultrasound
MSP	Mammography Screening Program
MX-only group	control group
MXUS	mammography and ultrasound
MXUS group	intervention group
PPV	positive predictive values
RCT	randomized controlled trial
RR	risk ratio
sIPTW	stabilized inverse-propensity treatment weighting
S-US	supplemental ultrasound
US	Ultrasound
