# Supplementary material for: Supplemental Breast Ultrasound in Mammography Screening for Women with Critically Dense Breasts
Source: Cancers (Basel). 2026 May 19;18(10):1631. doi: 10.3390/cancers18101631 (PMC13205084; doi:10.3390/cancers18101631)

Supplementary appendix

Supplement to:

Supplemental Breast Ultrasound in Mammography Screening for Women with Critically Dense Breasts

Heywang-Köbrunner Sylvia-H(1), Elsner Susanne(2), Haußmann Eva(1), Hacker Astrid(1), Grieger Paula(2), Hadwiger Moritz(2), Hertlein Michael(1), Katalinic Alexander(2)

(1) Referenzzentrum München
Sonnenstr. 29
80331 München

(2) Universität zu Lübeck
Institut für Sozialmedizin und Epidemiologie
Ratzeburger Allee 160
23562 Lübeck

Content

Supplementary tables 2

Table S1: Observed primary and secondary outcome measures 2

Table S2: Observed characteristics of the detected cancers 3

Supplementary figures 4

Figure S1: Covariate balance before and after sIPTW; A: Love plot of absolute mean differences for all covariates before and after weighting; B: Density plot of the propensity score for the MX-only group (Treatment = 0) and the MXUS group (Treatment = 1) before and after weighting. 4

Supplementary tables

Table S1: Observed primary and secondary outcome measures

	MXUS group (n = 25,341)	MX-only group (n = 38,529)	Absolute risk difference (ARD)	Relative Risk (RR)*
**Cancer detection rate**	N = 272	N = 277		
per 1000 [95% CI]	10·73 [9·46; 12·0]	7·19 [6·35; 8·03]	3·54 [2·02; 5·07]	1·49 [1·26; 1·76]
**Ductal carcinoma in situ rate**	N = 61	N = 85		
per 1000 [95% CI]	2·41 [1·80; 3·01]	2·21 [1·74; 2·67]	0·20 [-0·56; 0·96]	1·09 [0·79; 1·52]
**Invasive cancer rate**	N = 211	N = 192		
per 1000 [95% CI]	8·33 [7·21; 9·45]	4·98 [4·28; 5·69]	3·34 [2·02; 4·66]	1·67 [1·38; 2·03]
**Recall rate**	N = 1713	N = 2024		
in % [95% CI]	6·76 [6·45; 7·07]	5·25 [5·03; 5·48]	1·51 [1·13; 1·89]	1·29 [1·21; 1·37]
**Short-term follow-up rate**	N = 243	N = 190		
in % [95% CI]	0·96 [0·84; 1·08]	0·49 [0·42; 0·56]	0·47 [0·33; 0·60]	1·94 [1·61; 2·35]
**Biopsy rate**	N = 821	N = 551		
in % [95% CI]	3·24 [3·02; 3·46]	1·43 [1·31; 1·55]	1·81 [1·56; 2·06]	2·27 [2·04; 2·52]
**PPV1#**				
in % [95% CI]	15·88 [14·15; 17·61]	13·69 [12·19; 15·18]	2·19 [-0·01; 4·48]	1·16 [0·99; 1·35]
**PPV2§**				
in % [95% CI]	33·13 [29·90; 36·37]	50·27 [46·04; 54·51]	-17·14 [-22·47; -11·81]	0·66 [0·58; 0·75]

Legend: *Relative Risk for MXUS with reference MX-only, PPV positive predictive Value, #PPV1: proportion of detected cancers among participants with a positive finding following the consensus conference: §PPVII: proportion of detected cancers among those who underwent biopsy

Table S2: Observed characteristics of the detected cancers

	MXUS group (n = 272)	MX-only (n = 277)	P-Value
**Age**			0·390
Median [IQR]	56 [52–61]	55 [51–61]	
**DCIS, n (%)**	61 (22·4)	85 (30·7)	0·036
per 1000	2·41	2·21	
**Invasive breast cancers; n (%)**	211 (77·6)	192 (69·3)	0·036
per 1000	8·33	4·98	
**T stage, n (% of invasive cancer)**			0·173
T1	163 (77·3)	150 (78·1)	
per 1000	6·42	3·89	
T2	47 (22·3)	37 (19·3)	
per 1000	1·85	0·96	
T3/4	1 (0·4)	5 (2·6)	
per 1000	0·04	0·13	
**UICC stage, n (% of invasive cancer)**			0·376
Stage I	151 (71·6)	137 (71·4)	
per 1000	5·96	3·56	
Stage II	46 (21·8)	48 (25·0)	
per 1000	1·82	1·25	
Stage III-IV	9 (4·3)	6 (3·1)	
per 1000	0·36	0·16	
Stage missing	5 (2·4)	1 (0·5)	
per 1000	0·20	0·04	
**Histopathological grading, n (% of invasive cancer)**			0·735
Grade 1	63 (29·9)	49 (25·5)	
Grade 2	120 (56·9)	115 (59·9)	
Grade 3	17 (8·1)	19 (9·9)	
Grade missing	11 (5·2)	9 (4·7)	
**Invasive cancer size, mm**			0·732
Median [IQR]	14 [9-19]	14 [9-18]	

Supplementary figures

Figure S1: Covariate balance before and after sIPTW; A: Love plot of absolute mean differences for all covariates before and after weighting; B: Density plot of the propensity score for the MX-only group (Treatment = 0) and the MXUS group (Treatment = 1) before and after weighting.

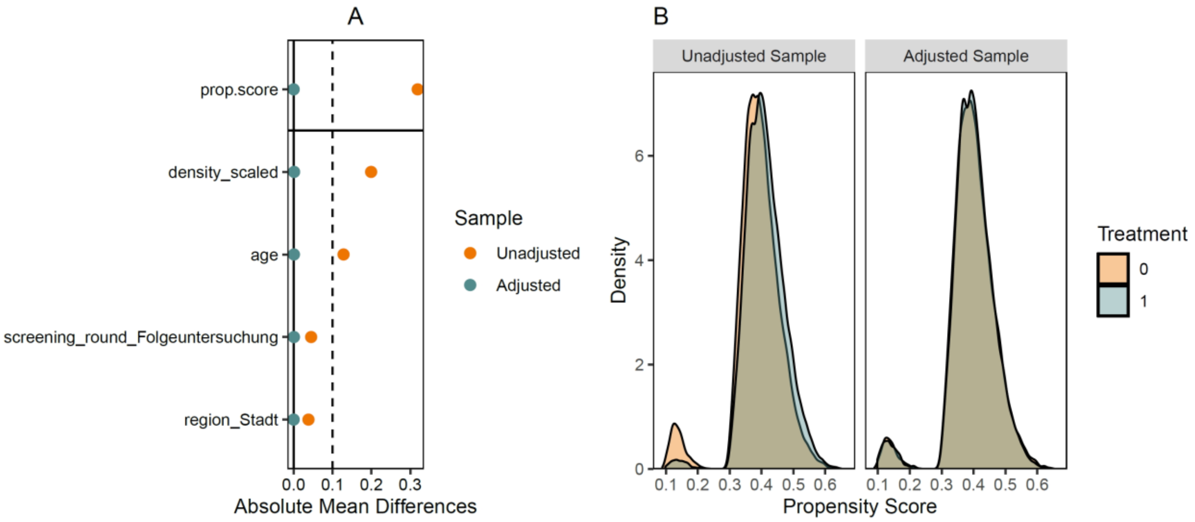

Supplement: Supplementary file 1 [file cancers-18-01631-s001.zip › cancers-4267419-supplementary.pdf]
